# The plurivocal university: Typologizing the diverse voices of a research university on social media

**DOI:** 10.1177/09636625241268700

**Published:** 2024-08-23

**Authors:** Sophia Charlotte Volk, Daniel Vogler, Silke Fürst, Mike S. Schäfer

**Affiliations:** University of Zurich, Switzerland

**Keywords:** decentralization, institutional communication, social media, plurivocality, science communication, scientists, social network analysis, Twitter, university communication

## Abstract

Science communication has diversified in the wake of the digital transformation of communication and media ecosystems. Social media enable universities, but also academics and institutions affiliated with them, to expand their communication. This leads to increasing plurivocality of universities, yet the many different voices remain largely unexplored. This study develops a typology to conceptually distinguish eight voices by their representational role, hierarchical embeddedness, type, and affiliation. Based on a quantitative content and social network analysis of more than 600 Twitter accounts linked to a research university, it identifies six types of voices empirically. The study compares interactions among these voices, showing differences between central and decentral, as well as institutional and individual voices, and highlighting closer exchanges between voices within the same disciplinary communities. It also examines topics and tonality, revealing that decentral institutional voices engage most in science-related topics, and that only current and former students express critical views.

## 1. Introduction

Science communication has diversified in recent years, mirroring the profound transformation of communication environments and the rise of digital media. Individual science communicators such as researchers have been incentivized and afforded opportunities to engage more in public communication, particularly with the emergence of social media (e.g. Jünger and Fähnrich, 2020; Koivumäki and Wilkinson, 2022; König, 2020). Part of this transformation is the rising importance of organizational science communication by research centers, scientific academies, or higher education institutions (Davies and Horst, 2016; Schäfer and Fähnrich, 2020). Especially universities, arguably *the* core institutions of the academic system, have professionalized and expanded their communication efforts, becoming increasingly relevant public communicators of science (Fürst et al., 2022a; Schwetje et al., 2020). This also extends to social media, where universities have created and professionalized official accounts to communicate about research, teaching, and organizational matters (e.g. Capriotti et al., 2023; Sörensen et al., 2023). Social media communication of scientific institutions represents a significant part of science communication online, often serving as a source for journalistic reporting, reaching audiences directly, and impacting the public’s perception of science (e.g. Brossard, 2013; Weingart, 2022).

The communication of universities in public online spaces is not only shaped by *official* accounts, however, but also—and probably even more so—by *other voices* that communicate on behalf of a university as well: departments, affiliated centers, labs, or individual researchers. Scholars assume that the diversity of voices on social media has increased due to the mediatization of science and competitive pressures on universities and academics (Davies and Horst, 2016; Thiele and Luethje, 2021; Weingart, 2022). Yet, the plurivocality of universities—across the diverse spectrum of individual and institutional voices—has hardly been explored. Examining the diversity of voices surrounding the public communication of universities is relevant to science communication research, because universities as key sites of knowledge production must fulfill a “third mission” of outreach and science communication (Fürst et al., 2022b) and because such voices can influence public attitudes toward universities and science more broadly (Väliverronen et al., 2022; Weingart, 2022).

To date, neither science nor organizational or strategic communication research has mapped the voices surrounding universities or analyzed their communicative interrelations in full. Most studies assessing university communication on social media focus on the official voice only—that is, one official account operated by communication professionals (e.g. Linvill et al., 2012; Palmer, 2013). Others analyze individual academics’ use of social media, their content, or engagement with followers (e.g. Jünger and Fähnrich, 2020). More recent studies shed light on social media communication by decentral units of universities like departments or research centers (Entradas, 2022). However, holistic analyses mapping and characterizing the entirety of voices and interactions of plurivocal universities on social media are lacking.

We provide such an analysis, focusing on one university and one social media platform. First, we conceptualize and typologize the multiple voices of a university based on their representational role, hierarchical embeddedness, type, and affiliation. Second, we present results of a quantitative content and network analysis of more than 600 accounts on Twitter linked to a typical research university.

## 2. University communication on social media

Social media have become important channels for a growing number of actors in science communication—from universities to funding agencies, journals, scientific associations, and academics to science influencers. Platforms such as X/Twitter, Facebook, Instagram, YouTube, LinkedIn, or TikTok are increasingly used for public science communication, but also for sharing information about jobs or conferences and networking within the scientific community (König, 2020), leading to blurring boundaries between public and intra-scientific discourses.

### Plurivocality of universities

Universities are often considered as *special* organizations. With their low degree of centralization and hierarchical governance, universities have been conceptualized as loosely coupled systems with little intra-organizational coordination between decentral units, such as faculties or departments (Krücken and Meier, 2006; Schwetje et al., 2020). Many individuals are only temporary members of universities—being students or scientific staff on temporary contracts—and academics often identify stronger with their scientific community than with their employer (Maassen and Stensaker, 2019).

Notably, universities are also polyphonic or plurivocal organizations (Hazen, 1993), harboring a multiplicity of voices that communicate relatively autonomously (Davies and Horst, 2016; Entradas, 2022). Unlike hierarchical organizations like corporations with top–down approaches to communication adhering to the ideal of “one voice,” academics in particular often communicate independently from their universities’ standpoint, and professional university communicators have limited control over and influence on them (Horst, 2013; Lo et al., 2019; Rödder, 2020). Studies indicate that some communication offices view a diversity of voices as a valuable extension of the official voice (e.g. Lo et al., 2019; Rödder, 2020), while others perceive a loss of power over how the university is represented on social media (Peters, 2022; Väliverronen et al., 2022). Professional university communicators have been shown to fear inconsistent and non-professional public communication, because other voices may not be sufficiently skilled to communicate or unwilling to adhere to organizational guidelines (Schwetje et al., 2020). The move away from “one voice” to plurivocality is often accompanied by communicators’ attempts to control public communication and to “orchestrate” voices to ensure “a harmonious choir with many voices but one melody” (Peters, 2022: 554). By providing social media training and guidelines, communication offices aim to enable university members to communicate professionally, thereby exerting indirect influence on otherwise autonomous subunits and individuals (Rödder, 2020; Rowe and Brass, 2011; Weingart, 2022). Naturally, tensions over the voice in plurivocal organizations can be expected (Väliverronen et al., 2022)—but research taking stock of this plurivocality is scarce.

### Conceptualization of voices

To map the diverse voices speaking in plurivocal universities on social media, we suggest using four analytical dimensions: representation, hierarchy, type, and affiliation.

- *Representation* refers to the permission and legitimacy granted to a particular voice to publicly communicate on behalf of—and thus officially represent—the *entire* university. This is typically mandated only to central communication departments and university leaders (rectors/presidents), who represent the university qua office.
- *Hierarchy* refers to the position of a voice within the organizational structure. Voices can be categorized as voices at the central level, meaning at the top of the hierarchy, or at (different) decentral levels (Entradas et al., 2024).
- *Type* describes the characteristic of voices and helps classify voices into institutional voices, representing departments or labs, or individual voices, which are reflected in personal accounts on social media (Debove et al., 2021).
- *Affiliation* focuses on the relationship of voices with the university, distinguishing between current members or organizational units, or former members, such as past employees or alumni.

These considerations give rise to a typology differentiating eight kinds of voices (Table 1).

**Table 1.** Systematization of voices in plurivocal universities.

Voices	Representation	Hierarchy	Type	Affiliation
(1) Official institutional voices (accounts of central communication department)	Yes	Central	Institutional	Current
(2) Official individual voices (official account of president)	Yes	Central	Individual	Current
(3) Central institutional voices (e.g. equal opportunity office, international office)	No	Central	Institutional	Current
(4) Central individual voices (e.g. spokesperson, head of international office)	No	Central	Individual	Current
(5) Decentral institutional voices (e.g. schools, departments, centers)	No	Decentral	Institutional	Current
(6) Decentral individual voices (e.g. professors, students)	No	Decentral	Individual	Current
(7) Former institutional voices (e.g. spinoffs)	No	Decentral	Institutional	Former
(8) Former individual voices (e.g. researchers, alumni)	No	Decentral	Individual	Former

In what follows, we briefly review the state of research on official, central, decentral, and former voices of a university on social media.

#### Official voices

Numerous studies have examined the *official institutional* voices, focusing on universities’ presence and content on social media (e.g. Bélanger et al., 2014; Linvill et al., 2012; Palmer, 2013). Universities often have official institutional accounts on Twitter, Facebook, Instagram, or LinkedIn (e.g. Capriotti et al., 2023; Sörensen et al., 2023), which are branded and recognizable as the official self-presentation (Alqahtani et al., 2020). These accounts are usually maintained by central communication offices, whose task is to safeguard reputation (e.g. Lo et al., 2019; Volk et al., 2023). Furthermore, university leaders such as presidents, who represent the university qua office, can communicate via official supra-individual accounts on social media, which we characterize as *official individual* voices. Such officially designated accounts—unlike individual personal accounts—can be co-managed by communication offices and passed on to the next president. To our knowledge, there are hardly any studies on this type of voice (but see Fürst et al., 2023 for university leaders’ social media orientation).

Beyond official voices, the various other—central, decentral, or former—voices of the university may be perceived as representations of a university (Peters, 2022), even if they are *not* authorized to act as official representations. Studies show that audiences perceive voices as voices of an organization if they have a publicly visible connection to this organization (Carr et al., 2023).

#### Central voices

Research on institutional or individual voices at the central level—beyond the official voice—is sparse.^1^ *Central institutional* voices belong to university-wide service units such as the alumni, gender equality, or international office, which, unlike the official voice, are not entitled to represent the entire university, but to speak about topics in their area (e.g. diversity, international exchange). Central units also maintain accounts on social media to reach their target groups, as the few existing studies have shown (e.g. Alqahtani et al., 2020). *Central individual* voices belong to individual members affiliated with central units, such as media spokespersons, heads of central offices, or administrative staff, who communicate on personal accounts—rather than officially designated accounts created qua position (Alqahtani et al., 2020; Kimmons et al., 2017). This can also apply to university presidents, rectors, or their deputies, who—given a trend to personalization on social media—may use their personal accounts.

#### Decentral voices

Most research has focused on individual rather than institutional voices at the decentral level. *Decentral institutional* voices belong to decentral units in universities, including departments, institutes, or research centers. They often have their own staff for communication, who are, however, not necessarily specialized in communication (Entradas, 2022) but legitimized to speak about their unit (Peters, 2022). Some decentral units engage in public communication quite independently from the central communication office and via their own social media accounts. Other decentral units rely on “the central communications offices to disseminate their news” (Entradas, 2022, p. 641), but large-scale studies are lacking. Much research has focused on *decentral individual* voices of a university, particularly academics who appear as experts and representatives of their university in public communication (Horst, 2013; König, 2020). Individuals at other career stages are hardly studied.

#### Former voices

These voices belong to former members of a university, for example, past employees or students, or formerly affiliated subunits (e.g. research centers, spinoffs) that have become independent from the university. Hardly any research exists on former voices, other than to suggest that alumni are often visibly connected to their alma mater via their personal social media profile or bio (Alqahtani et al., 2020; Knight and Kaye, 2016).

Collectively, these multiple voices configure the plurivocality of organizations on social media—or in our case, a university on Twitter.

Rebranded as X in 2023, Twitter^2^ has become one of the most popular platforms for scientific institutions and scientists: as of 2020, Twitter was used by an estimated number of nearly 300,000 scientists (Guenther et al., 2023; Zhang and Lu, 2022) and was the platform where universities were most active (Capriotti et al., 2023; Sörensen et al., 2023). Given its popularity in the scientific field, at least until Elon Musk’s purchase, we assume that Twitter is well-suited to study the plurivocality of universities. We aim to examine which of the conceptually derived voices can be found empirically on Twitter. We anticipate variations among voices in terms of reach (number of followers) and activity (number of tweets), with the official voices likely leading. Our first research question asks:

RQ1: Which voices can be identified in a plurivocal university, and how can they be characterized in terms of reach and activity?

### Interactions between voices

Twitter allows users to interact on multiple levels (Bruns and Moe, 2014), including the formation of follower–following networks, exchanges through #hashtags, interactions through likes or retweets, and conversations through @mentions or @replies. However, the interactions of voices within a given university have hardly been explored and two gaps remain.

First, there is scarce research on how official voices interact with different voices of the university. A study of official Twitter accounts of Saudi Arabian universities found that they often interact via mentions with other accounts, such as administrative bodies, departments, or the rector of their own university (Alqahtani et al., 2020), which represent central and decentral institutional voices in our typology. A study of all Swiss universities concluded that most mentions in social media posts by official accounts refer to internal actors (Sörensen et al., 2023), which were not specified, however. Moreover, a large-scale study of Twitter accounts speaking on behalf of U.S. universities revealed that most tweets were monologic rather than dialogic, indicating a low emphasis on interactions (Kimmons et al., 2017). The focus on one-way communication is also mirrored in other studies of official voices (e.g. Bélanger et al., 2014; Linvill et al., 2012), and somewhat striking given the affordances provided by Twitter as a social network. Quite similarly, studies of academics’ engagement on Twitter, including mentions, also indicate that interactions are rather low to moderate (e.g. Guenther et al., 2023). Since research remains sparse, specifically on the interactions of the multiple voices with the *official voices*, we address the following research question:

RQ2: How do the multiple voices interact with the official voices on Twitter through replies, mentions, and retweets?

Second, the voices of a university are embedded in larger networks and not only interact with the official voice but also among each other (Schwetje et al., 2020; Sörensen et al., 2023) and with other universities (Barnett et al., 2014). But social network analysis has rarely been applied to university communication on Twitter, and if so, with a focus on official accounts. A network analysis of the official accounts of Australian universities showed strong geography-based follower–following connections between universities (Palmer, 2016), while a co-mention network analysis of Saudi Arabian universities revealed that official accounts often engaged in Twitter conversations with accounts from within the same university (Alqahtani et al., 2020). While there are many studies examining academics’ networks on Twitter (e.g. Ke et al., 2017), their embeddedness in university communication is usually not investigated. Rather, most studies focus on hashtags or interactions with other academics or non-academic publics such as journalists (e.g. Walter et al., 2019). However, a survey among academics showed that they use Facebook for social networking and interaction with students or alumni (Roblyer et al., 2010). Given the lack of social network analyses, we aim to explore the conversations via mentions among the multiple voices on Twitter, assuming differences between voice types (Xu and Zhou, 2020). We ask:

RQ3: How do the voices of a plurivocal university interact with each other through mentions?

### Topics and tonality

Finally, we are interested in how science is communicated on social media. Different voices may emphasize different topics, that may or may not be related to research, and speak in different tones. To date, however, scholars have not compared the topics and tonality of the content communicated by different voices surrounding a university.

Most research has examined the topics of university’s official voices, often distinguishing academic content—such as research and teaching—and content that relates to the organization—such as its performance in rankings, personnel, finances, or sustainability (e.g. Capriotti et al., 2023; Sörensen et al., 2023). While the former topics represent public science communication, the latter rather serves “branding” or self-promotional goals of the university (Koivumäki & Wilkinson, 2020). Regarding the share of academic- versus organization-related content, studies have produced mixed findings. A study of official social media accounts of 70 universities found that organization-related posts accounted for roughly two-thirds, while academia-related content only made up one-third (Capriotti et al., 2023). A study of the official accounts of all Swiss universities contrarily revealed a rather similar ratio of organization- to academia-related content (Sörensen et al., 2023). In terms of tonality, most content posted on official accounts is positive or neutral (Kimmons et al., 2017). Overall, findings suggest that universities use official accounts rather for self-positioning than for engaging the public (Palmer, 2013; Sörensen et al., 2023)—but it remains unclear whether the other voices have a different focus.

While research has largely neglected content produced by other voices surrounding a university, there are several analyses of what academics, i.e. decentral individual voices, tweet about. A recent study found that academia-related topics account for roughly 60%, followed by tweets unrelated to science (28%; e.g. politics), and finally, personal interests (13%) (Guenther et al., 2023). In terms of tonality, the same study found that more than half of the tweets were factual, and of the remaining most were positive. Other studies also concluded that academics’ tweets are thematically diverse, and—despite a focus on science—include non-science-related content (Jünger and Fähnrich, 2020; Zhang and Lu, 2022). A few studies explained academics’ emphasis on science as a result of pressures to build their scientific reputation and “brand” themselves (Knight and Kaye, 2016; Thiele and Luethje, 2021). In contrast, we know little about the topics of decentral institutional voices. A study of a political science department’s Facebook fan page (Waite and Wheeler, 2014) revealed a mix of topics, including academia-related topics (e.g. department news) and non-science-related topics (e.g. political news). A cross-country survey indicated that decentral university units may engage more in science communication activities than central communication offices, perhaps because of their closer proximity to actual research (Entradas et al., 2024), but content analyses are lacking.

Against this backdrop, we aim to explore differences in topics and tonality between the voices when they engage in conversations via mentions with the official voices. Moreover, we aim at a comparison, assuming that official voices might focus most on organization-related topics, and decentral voices more on academia-related topics. Our last research question asks:

RQ4: What differences in topics and tonality emerge between voices when interacting via @mentions with the official voices, and how does this differ from the topics communicated by the official voices?

## 3. Method

We analyzed Twitter communication surrounding a research university—the University of Zurich—from Switzerland as a case study. The university has seven faculties and over 150 departments, roughly 28,000 students, and over 7000 employees. It is ranked among the top universities in Europe and embedded in a globally oriented higher education landscape. The university represents a quite typical case for a Western higher education system shaped by New Public Management (NPM) reforms (Davies and Horst, 2016; Fürst et al., 2022a). Analyzing Twitter makes sense because it is widely used among Swiss universities (see SM1 in the Supplemental Material; Sörensen et al., 2023).

Data collection took the university’s official institutional voices as a starting point. We retrieved a list of all followers of the university’s two *official accounts* (Table 2) through the Twitter application programming interface (API) and downloaded all tweets (original tweets, retweets, and replies) posted in 2021. We only included followers who (1) had mentioned or tagged the University of Zurich in their account’s self-description^3^, (2) were active in 2021 (i.e. had tweeted at least once), and (3) did not restrict access to their account.

**Table 2.** Characterization of voices.

Voice type	Account type	*n*	Since	Followers	Tweets
OFFICIAL VOICES					
Institutional	English language	1	01/2012	15,108	450
	National language	1	10/2011	21,512	673
Individual	Representative	–	–	–	–
	N	2	-	36,620	1123
Voice type	Account type	*n*	% of all 619 voices	*M* followers	*M* tweets
CENTRAL VOICES					
Institutional	Administrative body	4	0.6	835	285
Individual	Representative	11	1.8	303	131
	N_central_	15	2.4	445	172
DECENTRAL VOICES					
Institutional	Department/institute	31	5.0	1321	195
	Division/team/lab	18	2.9	822	145
	Administrative body	4	0.6	258	61
	Student body	6	1.0	523	74
	N_institutional_	59	9.5	1015	158
Individual	Professor	68	11.0	1492	259
	Researcher	100	16.2	667	178
	Postdoc	79	12.8	464	136
	PhD student	139	22.5	191	75
	Student	54	8.7	255	126
	N_individual_	440	71.1	557	144
	N_decentral_	499	80.6	611	146
FORMER VOICES					
Institutional	Unit	-	-	-	-
Individual	Employee	40	6.5	672	127
	Student	39	6.3	542	91
	N_former_	79	12.8	608	109
NON-IDENTIFIABLE		26	4.2	233	169
	N	619	100	591	148

For RQ1, the first two authors manually coded the self-descriptions of all 619 follower accounts (1.7% of all follower accounts) to identify whether they qualified as a voice, and then assigned them to different voice categories (see Table 2). In case of doubt, we searched the account on Twitter or used Google to verify that we had identified and categorized the account correctly. We also coded the predominant discipline (see the codebook part I in SM2 and Table S1 in the Supplemental Material).

To answer RQ2, we downloaded all tweets from the two official accounts of the University of Zurich for the same year (*n* = 1123; see Table 2). We then identified all tweets with an @mention of one of the two official accounts of the university (*n* = 2222) with an automated procedure using a regular expression (#\\w+) for capturing mentions. We also identified @replies to the official accounts (*n* = 19) and retweets of tweets of the official accounts (*n* = 519) by the multiple voices from the data retrieved from the Twitter API.

For RQ3, we investigated the @mention network between the identified voices using Gephi software. Mention networks are among the most common types of conversational networks formed by Twitter users (Xu and Zhou, 2020) and reveal patterns of frequent user interactions. To reconstruct the network, we selected all accounts which mentioned at least one other account of the University of Zurich in one of their tweets (371 accounts with 1465 directed @mentions). The accounts were organized into distinct communities using the Louvain algorithm for community detection (Blondel et al., 2008) based on account homophily. The concept of homophily describes the tendency of individuals to like and interact with other individuals if they perceive themselves to be similar to them, and has been identified as constitutional for social networks (McPherson et al., 2001). We chose the Louvain algorithm for the analysis because it is among the fastest and best performing community detection algorithms and performs well on small to mid-scale networks like ours (Anuar et al., 2021; see also Arlt et al., 2019). Our analysis revealed 11 communities (see section 4.2).^4^ The network exhibited a density of 0.012, denoting that only 1.2% of all potential connections between nodes were realized. Despite the dense interconnections in the follower network, we identified meaningful structural patterns, as substantiated by a modularity score of 0.49. The modularity score ranges from −0.5 to 1 and indicates how dense the connections within the communities are. Networks with a high modularity score have numerous connections within a community while showing sparse connections outward to other communities (Jacomy et al., 2014). Thus, high modularity means that community detection has successfully grouped nodes into high-density communities (Rustamaji et al., 2024).

For RQ4, we employed manual content analysis of tweets mentioning the official voices (*n* = 2222), and examined the tweets posted by the official voices (*n* = 1123). We coded the topics, tonality, and discipline by adapting existing codebooks (see codebook part II SM2 in the Supplemental Material). We coded three topical areas: academic (e.g. research, teaching), organizational (e.g. finances, personnel, alumni), and other topics (e.g. trivia, personal communication). For tonality, we assessed whether tweets were positive (e.g. praise), neutral (e.g. factual), or negative (e.g. criticism). Following coder training, all tweets were coded by a trained student coder. Intercoder reliability was measured for a random sample of 100 tweets, which was coded by the first two authors and the student coder. Krippendorff’s alpha delivered satisfactory results (topics α = .779, tonality α = .786, discipline α = .768).

## 4. Results

### Mapping and characterization of voices

Our analysis reveals a multiplicity of voices speaking in the case university’s name beyond the official voices, confirming the plurivocality of universities (Table 2). Out of eight conceptually derived voices, six—including the official institutional accounts—were empirically observed and further differentiated inductively.

A vast majority of accounts are *decentral voices* (80.6%), with most belonging to *decentral individual voices* (71.1%). They consist of PhD students (22.5%), researchers^5^ (16.2%), postdocs (12.8%), professors (11.0%), and current students (8.7%). The remaining accounts belong to *decentral institutional voices* (9.5%), which mostly belong to departments (5.0%), divisions, teams or labs (2.9%), decentral administrative bodies (0.6%), or student bodies of a faculty (1.0%).

In contrast, *central voices* make up only a very small fraction of all accounts (2.4%). Most belong to *central individual voices*, representing the personal accounts of the university’s president, spokespeople from the communication office, or heads of central service departments (1.8%). Only four accounts (0.6%) were identified that represent *central institutional voices* like the university’s equal opportunity office, the alumni relations office, or graduate career office. We did not identify any *official individual voices*, that is, officially branded accounts of university leaders.

In addition, we found accounts belonging to *former individual voices* (12.8%), which self-described as former researchers or alumni and may still be perceived as representatives of the university.^6^ We did not identify any *former institutional voices*. Finally, 4.2% of accounts could not be clearly identified as a distinct group.

We further characterized the multiple voices in terms of reach (manifested in average follower count) and activity (manifested in average number of tweets in 2021) (see Table 2). Institutional voices have a large reach, with *decentral institutional voices* having most followers on average (*M* = 1015), followed by the four *central institutional voices* (*M* = 835). Yet, professors have the highest follower number (*M* = 1492) among central and decentral voices; individuals’ reach tends to decrease with lower academic status. We also aggregated the follower numbers of the voices: while the two official accounts reach a total of 36,620 followers, the *decentral individual voices* combined have a total of 245,001 followers and the *decentral institutional voices* have a total of 59,910; in comparison, the *central voices* reach only 6677 followers combined. This illustrates the weight of *decentral voices* in the university’s outreach.

In terms of activity, institutional voices produce more content on average. *Central institutional voices* are most active and post 0.8 tweets per day (*M* = 285), followed by *decentral institutional voices* with 0.4 tweets per day (*M* = 158). Among the *individual voices*, professors and researchers were most active, with an average of 259 and 178 tweets, respectively, which equals 0.7 and 0.5 tweets per day. In comparison, the national language *official* account posts 1.8 tweets per day. However, in sum, *decentral voices* create much more tweets combined (*n* = 72,691) than the *central voices* combined (*n* = 2583), and both types produce much more content than the two *official voices* (*n* = 1123).

### Interactions and mention networks between voices

Next, we analyzed how the multiple voices interact with the official accounts of the case university (RQ2) and among each other (RQ3). First, there is very little interaction of the multiple voices via @replies to (*n* = 19) and retweets of (*n* = 519) the content posted on the official accounts by the central communication department. Conversely, however, the voices use @mentions of the official accounts (*n* = 2222) quite often in their own content.

For retweets of content posted by the official voices, findings show that 75.2% come from *decentral voices* and 21.4% from *central voices* (Table 3). Considering the low share of *central voices* (2.4%), they retweet at a much higher rate: the four accounts belonging to *central institutional voices* account for 12.7% of all retweets. Similarly, relative to their share of accounts (9.5%), *decentral institutional voices* also create a considerable part of all retweets (32.4%), with the 31 accounts belonging to departments or institutes retweeting the most (27.9%). In absolute numbers, most retweets come from *decentral individual voices* (42.9%). Among individuals, professors show the most retweets of all groups (19.5%), but the 11 accounts belonging to *central individual voices* (e.g. president, spokespersons) retweet proportionally more (8.8%).

**Table 3.** Interaction of multiple voices with the official voices.

Voice type	Account type	*n*	Mentions of official accounts		Retweets of official accounts	
CENTRAL VOICES			*n*	%	*n*	%
Institutional	Administrative body	4	238	10.7	65	12.7
Individual	Representative	11	68	3.1	45	8.8
	N_central_	15	306	13.8	110	21.4
DECENTRAL VOICES						
Institutional	Department/institute	31	427	19.2	143	27.9
	Division/team/lab	18	129	5.8	15	2.9
	Administrative body	4	8	0.4	7	1.4
	Student body	6	45	2.0	1	0.2
	N_institutional_	59	609	27.4	166	32.4
Individual	Professor	68	545	24.5	100	19.5
	Researcher	100	382	17.2	80	15.6
	Postdoc	79	108	4.9	14	2.7
	PhD	139	155	7.0	23	4.5
	Student	54	19	0.9	3	0.6
	N_individual_	440	1209	54.4	220	42.9
	N_decentral_	499	1818	81.8	386	75.2
FORMER VOICES						
Institutional	Unit	–	–	–	–	–
Individual	Employee	40	17	0.8	5	1.0
	Student	39	72	3.2	2	0.4
	N_former_	79	89	4.0	7	1.4
Non identifiable		26	9	0.4	10	1.9
	N	619	2222	100	513	100

For mentions of the official accounts in content produced by the multiple voices, the pattern is similar (Table 3): although *decentral voices* account for the majority of mentions of the official accounts (81.8%), *central voices* mention the official voices proportionally more often, accounting for 13.8% of mentions. Again, institutional accounts mention the official accounts proportionally more than individual accounts, both at the central and decentral levels. However, in absolute numbers, most mentions stem again from *decentral individual voices* (54.4%), particularly professors (24.5%). Among the *decentral institutional voices*, departments and institutes stand out and account for 19.2% mentions of the official accounts, even though their share is only 5% of all accounts. Thus, proportionally more interactions with official voices occur at the decentral level the higher the accounts are located in the university hierarchy, both for institutional and individual accounts.

**Figure 1.**
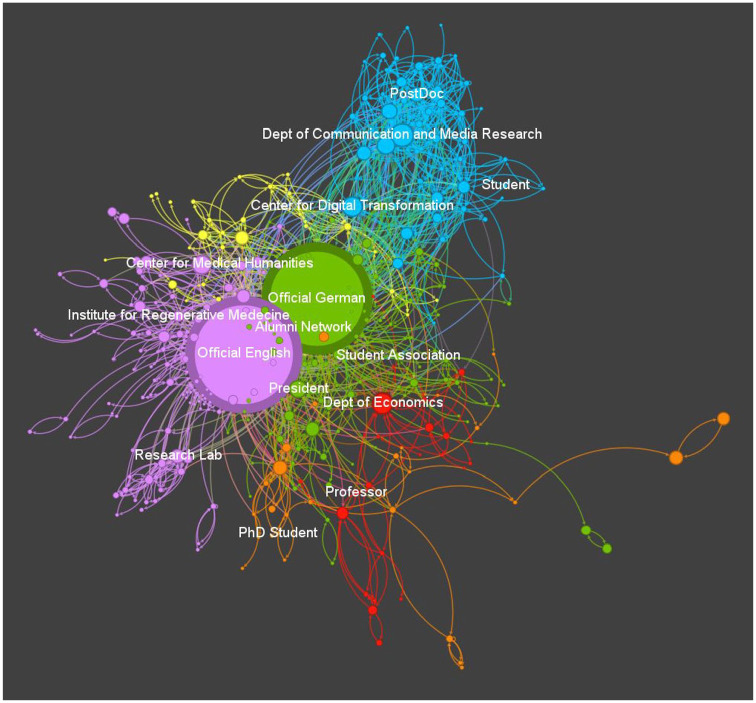
Interactions among all voices in the mentions network. *Note.* The nodes are color-coded to represent different communities: Medicine = violet, Central voices = green, Social Sciences = blue, Humanities = yellow, Linguistics = orange, and Economy = red. Exemplary accounts for communities and voice types are labeled. The visualization of the network was created using the ForceAtlas2 algorithm (Jacomy et al., 2014) in Gephi. The algorithm is force directed: nodes repulse each other while edges attract their nodes. Over a multitude of iterations this process leads to dense and stable clusters of nodes (accounts) where many edges (interactions through mentions) exist. Note that vertex size of the nodes is proportional to the indegree (i.e. large nodes represent accounts with many interactions through mentions). Please see online version for color figure.

Next, we explored how the multiple voices of the university interact with each other through @mentions, and what communities of voices with frequent interactions can be detected (RQ3). The analysis revealed six meaningful communities (see Table S2 in the Supplemental Material), which can be visualized as a network of mentions (Figure 1):

- Medicine (violet), *n* = 106 (28.6%)
- Central voices (green), *n* = 103 (27.8%)
- Social Sciences (blue), *n* = 56 (15.1%)
- Humanities (yellow), *n* = 28 (7.5%)
- Linguistics (orange), *n* = 28 (7.5%)
- Economy (red), *n* = 23 (6.2%)

Except for one large community—the *central voices* (green)—all communities are strongly driven by the discipline of the voices. Thus, the mention network shows that interaction through mentions mainly happens intra- and not inter-disciplinarily. Regarding the position of voices in the network, the algorithm assigned the two *official accounts* to two different communities, but close to each other. The national language account belongs to the first community of *central voices* (including, e.g., the president) and is more central in the network than the English account, which was assigned to the *medicine community*. This might be an effect of the high significance of this discipline during the COVID-19 pandemic, which led to interaction between individual academics and institutional accounts and the internationally oriented official English account.

We also examined how often the voices were mentioned (indegree), represented by the size of the nodes in Figure 1 and the eigenvector centrality, which shows how important a voice is in the network (for scores and details on eigenvector centrality, see Table S2 in the Supplemental Material). As expected, and also due to the sampling procedure, the two *official accounts* were mentioned the most. The *social science community* has the lowest share of connections to the other communities (15.6%) and appears more peripheral in the network (see Table S2 in the Supplemental Material). However, it also has the highest average indegree, indicating that voices of this community were often mentioned by other members of the community. Other communities have larger accounts, sometimes bridging the communities to the *official accounts*, like the economy department. The *central voices* have the second highest share of external connections (35.0%), indicating that the community is comparably well connected with other communities. Only the small *humanities community* has a higher share of external connections (40.5%).

### Topics and tonality of voices

Finally, we investigated what topics the voices communicate about in their tweets that mention the official voices (*n* = 2222), and then compared how the topics differ from the tweets (*n* = 1123) produced by the official voices (RQ4).

Results reveal considerable differences among the voices (Table 4). When mentioning the official accounts, *decentral voices* focus less on organizational (20%) and much more on academic topics (60%) than *central voices*, which almost equally emphasize both academic (47%) and organizational topics (42%). The highest amount of academic content is posted by *decentral institutional voices* (72%). Mentions related to organizational topics at the decentral level focus mostly on job opportunities, showcasing achievements, or social responsibility. At the central level, the focus of organizational topics is mostly on alumni relations, support for early career researchers, or grant opportunities. When *central voices* mention the official accounts with regard to academic topics, they often tweet about upcoming events or findings from funded research projects.

**Table 4.** Topical areas in mentions of official voices by the multiple voices.

Voice type	Account type	Amount of mentions	Topics in %			Sentiments in %		
			Academia	Organization	Other	Positive	Neutral	Negative
CENTRAL VOICES								
Institutional	Administrative body	238	50	47	3	18	82	0
Individual	Representative	68	38	24	37	31	66	1
	N_central_	306	47	42	10	21	78	0
DECENTRAL VOICES								
Institutional	Department/institute	427	74	21	5	21	78	0
	Division/team/lab	129	74	15	2	26	72	0
	Administrative body	8	75	25	0	0	100	0
	Student body	45	47	40	13	18	82	0
	N_institutional_	609	72	21	7	22	77	0
Individual	Professor	545	54	19	26	27	70	2
	Researcher	382	54	20	26	29	69	1
	Postdoc	108	54	20	26	35	62	3
	PhD	155	61	19	20	28	70	2
	Student	19	32	42	26	16	58	26
	N_individual_	1209	55	20	25	28	69	2
	N_decentral_	1818	60	20	19	26	72	2
FORMER VOICES								
Institutional	Unit	–	–	–	–	–	–	–
Individual	Employee	17	35	18	47	24	76	0
	Student	72	25	60	11	8	63	25
	N_former_	89	27	52	18	11	65	20
Non-identifiable		9	67	0	22	11	78	0
	N	2222	57	24	18	25	73	1

*Note.* In a few cases, topics or tonality were not identifiable or not applicable (e.g. in tweets with only mentions but no text), resulting in a few missings, which is why the percentages do not always add up to 100.

Individual accounts at both central and decentral levels mention the official accounts also in relation to other content, which often includes interpersonal communication (replies with mention) such as congratulations or thank-you notes: for *decentral individual voices*, this is the case in 25%; for *central individual voices*, it is even the case in 37%. Interestingly, there are few topical variations among *decentral institutional* and *decentral individual voices*, except for one group: students. The share of mentions related to organizational topics is quite high both for student bodies (40%), current students (42%), and former students (60%). Former students mostly tagged the university’s official accounts in tweets regarding the COVID-19 pandemic and the measures taken by the university against it.

A closer look at the tonality reveals that current and former students are the only voices with a substantial proportion of negative evaluations (25% and 26%, respectively). They often voice criticism in their mentions of the university’s official accounts, but the majority received neither likes nor retweets, and thus got very little attention. Notably, these negative mentions stem from only five accounts, one of which created the majority of critical tweets (almost all about the university’s pandemic measures). Beyond student tweets, mentions of the official accounts are largely neutral and sometimes positive in tone. Negative mentions are created only from accounts of individuals, and not from institutional accounts. Regarding positive mentions, these quite often coincide with what was coded as other content, that is, congratulations or thank-you notes, followed by research-related content.

Finally, we compared the topics in mentions of the official voices (*n* = 2222) with the topics in tweets produced by the official voices (*n* = 1123). The multiple voices combined mostly mention the official accounts in tweets related to academic content (57%), followed by organizational content (24%), and other content (18%) (Table 4). Surprisingly, the distribution is hardly different for the two official accounts (see Table S3 in the Supplemental Material): Tweets produced by the communication department contain slightly more academic (62%) and organizational content (27%), and less other content (11%). A closer look at the topics coded within academic content reveals that the focus of both the university’s official voices and the multiple voices is on research^7^ (e.g. publications) (45% vs 33%), events (e.g. announcement of scientific conferences) (14% vs 21%), and finally teaching (3% vs 4%).

There are no differences when comparing overall engagement rates (likes and retweets combined) (see Table S3 in the Supplemental Material). Yet a closer look at the sub-topics and their engagement metrics reveals some differences. For academic topics, teaching-related tweets by the multiple voices achieved higher engagement rates (*M* = 17.4) than those by the official accounts (*M* = 8.7). For organizational topics, official voices’ health-related tweets amid COVID-19 received most engagement (*M* = 30.3). For the multiple voices, job announcements created the highest engagement (*M* = 38.7), followed by tweets on performance (*M* = 15.8).

## 5. Discussion

Our analysis shows considerable diversity among the voices of the case university, the University of Zurich. Together, the 619 identified voices create much more content, reach, and engagement than the university’s two official accounts—although not all voices contribute equally, with a small number of professors and departments showing high activity. Interaction patterns show that the different voices interact with the official accounts through mentions or retweets to a moderate degree, reminiscent of a predominantly unidirectional mode of communication (Kimmons et al., 2017). Network analysis further reveals that conversations via mentions remain mostly within disciplinary boundaries, supporting the relevance of scientific communities (Maassen and Stensaker, 2019) and previous research indicating homophily in Twitter networks (Xu and Zhou, 2020). Content analysis shows that the different voices communicate quite often about organizational topics in either a neutral or positive tone, and thus contribute to a positive image of the university. Hence, and contrary to fears of professional university communicators that social media might negatively affect the university image (Peters, 2022; Schwetje et al., 2017), the identified lack of critical conversations indicates a low risk of reputational damage. On the contrary, the self-representation of the university is more likely to be strengthened—at least in this particular case. In the following, we contextualize our findings in light of previous research.

- *Official voices* of the case university, surprisingly, employ Twitter mainly for science communication, producing a larger share of academic content (62%, with 45% research-related posts) than self-promotional organizational content (27%). This is contrary to findings from previous studies of official social media accounts (Capriotti et al., 2023) and might be a typical feature of Swiss research universities (Sörensen et al., 2023).
- *Central voices* (2.4%) constitute only a modest fraction of the entirety of voices surrounding the university and comprise both institutional and individual accounts, including the personal account of the president or media spokespersons—which may be perceived by audiences as official representations (Carr et al., 2023). Central voices exhibit a comparatively high level of interaction with the official voices, reflecting their proximity in the university hierarchy. Central institutional accounts post almost equally about academic (50%) and organizational topics (47%), thus contributing comparatively more to reputation-building efforts than other voices.
- *Decentral voices* (80.6%) form most of the identified voices and comprise both institutional accounts (9.5%) and individual accounts (71.1%). Overall, decentral accounts contribute more tweets and boast a broader reach compared with central and official voices, suggesting that they play a pivotal role in shaping the university’s public representation, potentially even eclipsing the official accounts in terms of influence. Professors have the highest average follower count among all voice categories. Generally, the higher the position of decentral voices in the university hierarchy, the more active these voices are in producing content and engaging with the official voices. Decentral institutional voices stand out for their substantial focus on academic content (72%), aligning with the decentralization hypothesis (Entradas, 2022). Decentral individual voices, in contrast, tend to focus on academic topics (55%) but also engage in personal conversations (Guenther et al., 2023), reflecting academics’ “muddled” identities on social media (Koivumäki & Wilkinson, 2020). Interaction of individual accounts with the official voices is lower, possibly indicating a stronger affiliation with the decentral level than the entire university. Notably, enrolled students are the only currently affiliated voice publicly criticizing the university; voicing criticism of their employer on social media might be difficult for academics and other administrative staff (Knight and Kaye, 2016).
- *Former voices* (12.8%) comprise former employees and alumni who—due to their independence from the university—may more likely take a critical stance toward it.

## 6. Conclusion

Social media has become increasingly important for science communication with the public (e.g. Brossard, 2013), particularly for scientific institutions like universities (e.g. Sörensen et al., 2023). But little is known about the different voices communicating online on behalf of universities. We address this gap by providing the first holistic analysis, examining more than 600 voices surrounding one case university on Twitter.

Our study contributes to scholarship in several ways. First, we introduce a typology for categorizing eight different voices in university communication based on their representational role, hierarchical embeddedness, type, and affiliation, and propose an operationalization. Conceptually and methodologically, this approach can be used in follow-up studies to examine other organizations on social media. Second, we make an empirical contribution by reconstructing a case university’s communication on Twitter to identify and characterize six of these voices. Results indicate a diversification of voices on social media and illustrate the plurivocality of universities (Väliverronen et al., 2022). We demonstrate the relevance of individual and institutional voices at the decentral level, providing support for the decentralization hypothesis (Entradas, 2022), and shed light on central and former voices for the first time. Results emphasize the need to include such diverse voices in future studies that intend to reconstruct online communication of science and scientific institutions (or organizations more generally) comprehensively. Third, we demonstrate differences in the reach and activity of different voices, their interactions, and content. Our results align with research showing a rather low level of interaction and a dominance of positive and neutral tweets (Guenther et al., 2023), suggesting that the potential of social media platforms for engaging in dialogical and critical discourses about science and scientific institutions is probably not exhausted (Kimmons et al., 2017).

Overall, our study contributes to a more comprehensive understanding of the diverse voices representing science and universities in public online spaces (Brossard, 2013; Schäfer and Fähnrich, 2020). Still, it has limitations. It focuses on one university and platform only, at a certain point in time (during COVID-19), limiting its scope and making it harder to assess the generalizability of its results to other universities or scientific organizations, or to other platforms like LinkedIn or Instagram. Nevertheless, we assume that the same types of voices are found in other organizations, but that their relative distribution, topical focus, and tonality might differ—probably also depending on the platforms that serve different audiences.

Furthermore, due to our sampling strategy, we may have overlooked accounts not explicitly mentioning the analyzed university at the time of data collection, as well as accounts not following the university’s official accounts. Moreover, because our content analysis focuses only on mentions of the official accounts and excludes the remaining tweets of all voices (as well as links, pictures, emojis, etc.), we cannot draw conclusions about the topics that the many voices communicate about when they do *not* mention the case university’s official account.

Although we were able to identify meaningful structures after intense manual validation, two potential limitations of our social network analysis should be taken into account. First, the Louvain algorithm maximizes modularity and may fail to detect small communities within larger communities (Rustamaji et al., 2024). As reaching an optimal solution for communities in a social network is a computationally challenging task, the algorithm uses greedy optimization techniques, that is, “shortcuts or rules of thumb to find a solution that is close to the optimal solution” (Rustamaji et al., 2024: 2), potentially limiting the quality of the community detection. Second, even though the Louvain algorithm is well-suited for analyzing small to mid-scale networks (e.g. Anuar et al., 2021) and in our case delivered meaningful results, it has been shown to have disadvantages in detecting communities in social networks, for instance, insufficient connections between communities (Traag et al., 2019).

Our typology of voices on social media, while perhaps not exhaustive, can nevertheless lay the foundation for future research. It applies not only to Twitter and the analyzed case of the University of Zurich, but also to other social media platforms and types of organizations, opening the door for comparative studies across voices, platforms, organizations, and countries. Large-scale follow-up studies could identify the diversity of voices and their representations of science—for example, for public versus private research institutions—and provide information on varying proportions of content representing academic versus organizational aspects. Moreover, population surveys could explore audience perceptions of the authenticity or credibility of decentral, central, and official voices as well as institutional and individual voices. With their characteristic decentralized and loosely coupled organizational structures, universities have been an ideal setting for the study of plurivocality. However, the findings also pave the way for research that transcends science communication and extends to other research fields such as political communication, journalism, or strategic communication. The diversity of voices could be studied for political parties, nongovernmental organizations (NGOs), or corporations, which exhibit different organizational characteristics, but also have decentral voices (e.g. politicians) that together with the official voice (e.g. the party) shape public perceptions. Future studies could identify whether embracing a diversity of voices versus exerting top–down control is dependent on certain organizational factors, for example, culture, centralization, or communication strategy (e.g. Brockhaus et al., 2020; Koivumäki and Wilkinson, 2022; Väliverronen et al., 2022), and explore the ways in which scientific institutions are indeed special organizations.

## Supplemental Material

sj-docx-1-pus-10.1177_09636625241268700 – Supplemental material for The plurivocal university: Typologizing the diverse voices of a research university on social media Supplemental material, sj-docx-1-pus-10.1177_09636625241268700 for The plurivocal university: Typologizing the diverse voices of a research university on social media by Sophia Charlotte Volk, Daniel Vogler, Silke Fürst and Mike S. Schäfer in Public Understanding of Science

## Notes

1. In the following, the term “central voices” always refers to central institutional and central individual voices, meaning the voices at the central level without an official representative function (“official voices”).

2. We stick with the term Twitter because the data are from 2021.

3. By using this as a sampling criterion, we missed accounts that did not connect to the case university through written text (e.g. using a logo in their Twitter profile picture). We assume that particularly accounts of academics are missing from the sample: although universities may ask academics to make their affiliation visible in their social media profile, indicating an affiliation is optional (Koivumäki and Wilkinson, 2020).

4. We excluded five very small user communities (a total of 22 accounts) that lacked any discernible connections within the broader network.

5. Such accounts did not contain any further description that allowed us to assign them to a specific career stage.

6. In the following descriptions of results, we do not put an emphasis on these former voices.

7. The official accounts mainly tweet about published studies by academics of the university, most often from the natural sciences (63%), followed by social sciences (20%) and humanities (9%). Moreover, they share media interviews with members from the university and information about research infrastructure (e.g. new laboratories).
