# Supplementary material for: The plurivocal university: Typologizing the diverse voices of a research university on social media
Source: Public Underst Sci. 2024 Aug 23;34(3):270–90. doi: 10.1177/09636625241268700 (PMC11927015; doi:10.1177/09636625241268700)
Supplement: sj-docx-1-pus-10.1177_09636625241268700 – Supplemental material for The plurivocal university: Typologizing the diverse voices of a research university on social media [file sj-docx-1-pus-10.1177_09636625241268700.docx]

**Supplemental Material**

**The plurivocal university:**

**Typologizing the diverse voices of a research university on social media**

Sophia Charlotte Volk, Daniel Vogler, Silke Fürst, Mike S.  Schäfer

University of Zurich, Switzerland

Table of contents

[SM1. Description of the case university 2](#_Toc169770155)

[SM2. Codebook 3](#_Toc169770156)

[Table S1. Characterization of voices by discipline 8](#_Toc169770157)

[Table S2. Communities of voices based on mentions 9](#_Toc169770158)

[Table S3. Comparison of topics between official voices and all the multiple voices 10](#_Toc169770159)

[References 11](#_Toc169770160)

# **SM1. Description of the case university**

The case university – the University of Zurich – is a full research university with seven faculties and over 150 institutes, roughly 28’000 students and more than 7’000 employees in Switzerland. It is frequently ranked among the top universities in Europe and embedded in a globally oriented higher education landscape known for innovative research. The case university launched a German-language Twitter account in October 2011 and an English-language account in January 2012. At that time, roughly half of the universities in Switzerland had joined Twitter (Sörensen et al., 2023). By the year of data collection in 2021, which was marked by the expiration of the Covid-19 pandemic and before Elon Musk’s acquisition of Twitter, more than 70% of the country’s universities had a Twitter account. Overall, we believe that the University of Zurich represents a suitable and quite typical case for research universities in Western higher education systems shaped by NPM reforms (Davies & Horst, 2016; Fürst et al., 2022).

# **SM2. Codebook**

**Part I: Content analysis of Twitter account descriptions**

| **Var 1 Hierarchy** | | |  |
| --- | --- | --- | --- |
| Nominal, categorial | | |  |
| Definition | The variable records where a social media account is located in the university hierarchy. | | |
| Conventions | - Social media accounts at the central level include all accounts belonging to units that are located at the top of the university hierarchy and offer university-wide services or perform university-wide roles (e.g. Equal Opportunities Office, International Office, central communications department); this also includes accounts of employees in central departments. - Social media accounts at the decentralized level typically include the accounts of departments, institutes, or research centers as well as all accounts of employees who do not work in the central departments. | | |
| Var | Code | | |
| Hier_1 | 1 | Central level |  |
|  | 2 | Decentral level |  |
|  | 99 | NA |  |

| **Var 2 Typ** | | |  |
| --- | --- | --- | --- |
| Nominal, categorial | | |  |
| Definition | The variable records which type a social media account belongs to. | | |
| Conventions | - Social media accounts can belong to institutional units - such as the administration, individual institutes, or laboratories - or to university members - such as professors, postdocs, or students. - "Administrative body" is coded if it is a unit with administrative tasks (e.g. teaching evaluation, graduate promotion), regardless of its position in the university hierarchy. - "Administrative employee" is coded if the account belongs to an individual with no research focus (e.g., head of a service unit, or with a representative function such as rector/president). - "Researcher" is coded if an assignment to other codes (e.g. postdoc, professor) is not possible, but a research activity is evident from the description. - "Student" is coded both for BA/MA students and is also coded if it concerns former students, i.e., alumni; the type of membership is recorded in Var 3. - Accounts are researched on the Internet if the assignment is not clear from the description. | | |
| Var | Code | | |
| Typ_2 | *Institutional type* | | |
|  | 1 | Administrative body |  |
|  | 2 | Department/institute |  |
|  | 3 | Division/team/lab |  |
|  | 4 | Student body |  |
|  | 5 | Other unit |  |
|  | *Individual type* | |  |
|  | 6 | Administrative employee (e.g., head of service department, incl., rector/president) |  |
|  | 7 | Professor |  |
|  | 8 | Researcher |  |
|  | 9 | Postdoc |  |
|  | 10 | PhD student |  |
|  | 11 | Student |  |
|  | 99 | NA |  |

| **Var 3 Membership** | | |  |
| --- | --- | --- | --- |
| Nominal, categorial | | |  |
| Definition | The variable records the membership relationship between a social media account and the university. | | |
| Conventions | - The social media accounts can indicate a current membership (e.g. Researcher @UNI) or a former membership (e.g. PhD from @ UNI, Alumnus @ UNI, Ex-@ UNI, Formerly Postdoc @ UNI). - "Current membership" is also coded if no temporal terms are used (e.g. "currently Postdoc", "ongoing PhD" etc.). In case of doubt, the placement of UNI in the account description (at the beginning/end) and possible mentions of other institutions are also taken into account. - In case of doubt, accounts are not researched. | | |
| Var | Code | | |
| Mem_3 | 1 | Current membership |  |
|  | 2 | Former membership |  |
|  | 99 | NA |  |

| **Var 4 Representation** | | |  |
| --- | --- | --- | --- |
| Nominal, categorial | | |  |
| Definition | The variable records whether a social media account appears as a representation of the entire university. | | |
| Conventions | - The authority to communicate publicly on behalf of the entire university (and not just individual units) and to represent it officially generally lies only with communication departments and the university management (Rector/President). In social media accounts, this is indicated by an explicit designation of the account as "official" (e.g. "Official Account of Uni"). - For social media accounts of heads of communication, rectors, or presidents, it must be checked in each case whether it is a personal account or an official account qua position (e.g. "Official Account of the Rector"), which can also be passed on to a successor, for example. - All other accounts have no official authority to represent the entire university and are therefore always coded as "No official representation of the entire university"; this does not require a search. | | |
| Var | Code | | |
| Rep_4 | 1 | Official representation of the entire university |  |
|  | 2 | No official representation of the entire university |  |
|  | 99 | NA |  |

| **Var 5 Discipline** | | |  |
| --- | --- | --- | --- |
| Nominal, categorial | | |  |
| Definition | The variable records the discipline to which a social media account is assigned. | | |
| Conventions | - The overwhelming majority of account descriptions mention only one discipline and are therefore unambiguous in their coding. For the remaining posts, either the primary or first discipline is decisive. - The discipline is always coded if possible; for Var 2, institutional accounts are checked; personal accounts are not researched. Personal accounts are not researched. | | |
| Var | Code | | |
| Disc_5 | 1 | Natural sciences (Maths, Physics, Biology, Chemistry, Medicine, Informatics, Robotics…) |  |
|  | 2 | Social sciences (Psychology, Sociology, Computational Communication Science, (Computer-)Linguistics, Law, Economics…) |  |
|  | 3 | Humanities (History, Literature, Digital Religions…) |  |
|  | 99 | NA |  |

**Part II: Content analysis of tweets and mentions**

For coding the topics, tonality, and discipline, we adapted existing codebooks developed and tested by the authors in previous studies (Fürst et al., 2021; Sörensen et al., 2023; Vogler, 2020).

| **Var 1 Topic** | | |  |
| --- | --- | --- | --- |
| Nominal, categorial | | |  |
| Definition | The variable captures what the main topic of the tweet is about. In doing so, the coders consider all textual and visual elements of the tweet. | |  |
| Var | Code | |  |
| Top_1 | 1 | **Scientific Research**  Research-focused content, e.g., research results, publications, scientific projects, and collaboration; report of past scientific conferences and meetings, research infrastructure, media interviews about research |  |
|  | 2 | **Scientific Performance and Achievements**  Information on rankings; awards (e.g., Prima); significant positions (e.g., Journal Editor) for current and former members within the scientific community; congratulations on successes such as securing grants, publications without additional content value |  |
|  | 3 | **Events or Conferences**  Announcements/Calls for upcoming events (e.g., Call for Papers) with invitations to participate, register, as well as award competitions; also includes public events, webinars, etc.  Note: no jobs (→ Code 5 Personnel), no funding calls (→ Code 4 Funding), no pure teaching events (→ Code 6 Teaching). |  |
|  | 4 | **Funding**  Service information about deadlines, calls for proposals, and funding lines (also for PhDs, e.g., FAN); investment of funds; negotiation of financial resources; tuition fees; funding problems; as well as information about research funding from third-party funders, e.g., SNF, InnoSuisse, and EU Horizon 2020. |  |
|  | 5 | **Personnel/Working at Uni**  Job advertisements; coverage of the rector or chairholders; recruitment; communication with employees; selection processes; further education opportunities (e.g., Teaching Skills, Career Success Stories); salary systems; work atmosphere and motivation as well as services for the benefit of the staff (childcare facilities, yoga, etc.).Formularbeginn |  |
|  | 6 | **Teaching and Students**  Study program including Master Days; student achievements and projects; stay abroad; awards for students (e.g., study prize); supervision of theses, software, hardware, library, student life, etc. |  |
|  | 7 | **Alumni and Career Outside of Science**  Information about careers outside of research, including announcements/congratulations to alumni on new significant positions, alumni trips, etc |  |
|  | 8 | **University Governance**  Governance-related contributions including committees, e.g., University Council; workshops and events focusing on the strategic organizational development of the university; annual reports |  |
|  | 9 | **University Politics**  Externally determined educational policies and regulations; e.g., disciplinary regulations. |  |
|  | 10 | **Politics**  Political initiative; public referendum or decision of the Federal Council and other political fields outside of university politics; e.g., guest lectures from politics on security policy. |  |
|  | 11 | **Social Responsibility and Scientific Ethics**  CSR, scientific, social, and ecological responsibility of the university (e.g., diversity, sustainability) as well as general scientific ethical questions (e.g., Open Science); public events and occasions that cannot be clearly categorized as culture or sports (e.g., Future Day, Scientifica). |  |
|  | 12 | **Economy**  Collaboration with economic actors; activities and events that support students in entering professional life; labour market; entrepreneurship (e.g., spin-offs like UNI Startup) |  |
|  | 13 | **Culture, Art, and Sports**  Holidays, e.g., Christmas, Halloween, or Valentine's Day; occasions such as International Women's Day; Sexual Harassment Day; World Science Day; anti-smoking campaigns, sports; public events and occasions related to culture and sports; including exhibitions in UNI museums. |  |
|  | 14 | **Health Services**  Information and services for combating Covid-19 (e.g., vaccination bus, mask mandate); not research; suicide, mental health, etc. | |
|  | 77 | **Other or not recognizable**  If none of the above codes apply, gossip and chit-chat, weather, service posts (e.g., server outage), private matters (sports), personal responses (like "congrats", "thank you", etc.) |  |
|  | 99 | **NA (only Mentions)** |  |
| Conventions | - For panels, keynotes, and book publications where results are presented or experts have their say, "Research" is coded. Exceptions are specific discussions of another topic such as funding or university policy. - If researchers present their own results, we code "Research". If a researcher from the university is called in as an expert, the specific topic to be discussed is coded (e.g., "Politics"). If the specific topic does not occur, we assign "Other". - University rankings should always be coded as "Scientific Performance and Achievements". - Achievements, awards, and congratulations addressed to members of the university (but not to students) are coded as "Scientific Performance and Achievements". - For posts about upcoming "Events or Conferences" (conferences, call for paper), the entire text (including below the image) that belongs to the post itself is coded. The link to the event page is explicitly not part of the post. - Organizations such as SNSF, Innosuisse and Horizon 2020 usually concern financing and are to be assigned accordingly to "Funding". - Study trips are generally coded as "Teaching and Students", unless the focus is explicitly on a different topic. Also, in the case of invited lectures, "Teaching and Students" is always assigned as long as no explicit invitation to the public or description as a public event. Performances by students are generally coded as "Teaching and Students" as well. - Tweets about sporting events (e.g. Sola), holidays and festivities such as Christmas, Halloween etc. should be coded as "Culture, Arts and Sports". Announcements about publicly accessible facilities such as theaters, museums and botanical gardens or gastronomy, art and the hotel industry are also coded as "Culture, Arts and Sports", unless they are explicitly research-oriented. If the function of a post is to encourage or invite society to participate, we also assign " Culture, Arts and Sports", as we do for open days. - University future days (e.g. Scientifica) are coded as "Social Responsibility and Scientific Ethics". - Events for students with career talks from former students that focus on their professional future is assigned to the code "Alumni and Career Outside of Science". - Contributions on the topic of collaboration with industry should be coded as "Economy". This is easily recognizable by industry collaborations. - Posts about gossip, memes, and GIFs as well as service announcements are to be assigned as "Other". | |  |

| **Var 2 Tonality** | | |
| --- | --- | --- |
| Nominal, categorial | | |
| Definition | The variable records whether the tweet contains positive, neutral, or negative tone. | |
| Conventions | - "Positive" is coded if, for example, advertising, self-promotional, positively connoted terms (in the sense of "promotion" or "self-marketing") are used. These must be manifest in the text. Possible terms would be e.g. "congratulations", "prize", "fantastic", "hope you have a great time". - "Neutral" is coded if the focus is on the classic communication of science without evaluation or self-presentation; the focus here is primarily on the content. Possible terms would be e.g. "Thank you", polite phrase "hope to see you soon". - "Negative" is coded if the post contains criticism. - "NA" is coded if the post contains no text but only mentions, for example. - When coding the tonality, the hashtags must also be taken into account; images are not relevant. | |
| Var | Code | |
| Ton_2 | 0 | Neutral (factual) |
|  | 1 | Positive |
|  | 2 | Negative (critical) |
|  | 99 | NA (only @mentions) |

| **Var 3 Discipline** | | |  |
| --- | --- | --- | --- |
| Nominal, categorial | | |  |
| Definition | The variable records which discipline is primarily mentioned in the tweet. | | |
| Conventions | - The overwhelming majority of tweets mention only one discipline and are therefore unambiguous in their coding. In the few remaining tweets with multiple disciplines, either the primary or first discipline is decisive. - Discipline is also assigned to topic categories that are not about research (e.g. events). - The discipline is always coded if possible (e.g. also for guest lectures, funding, etc.); institutional units are checked; personal accounts are not researched. - If it is an interdisciplinary research project in which various disciplines are involved, it is coded as "Interdisciplinary". - In a few cases, no discipline will be recognizable (grants, diversity, etc.). - When coding the discipline, the hashtags or mentions must also be taken into account; images are not relevant. | | |
| Var | Code | | |
| Disc_3 | 1 | Natural sciences (Maths, Physics, Biology, Chemistry, Medicine, Informatics, Robotics…) |  |
|  | 2 | Social sciences (Psychology, Sociology, Computational Communication Science, (Computer-)Linguistics, Law, Economics...) |  |
|  | 3 | Humanities (History, Literature, Digital Religions…) |  |
|  | 4 | Interdisciplinary (e.g., NCCR, UFSP) |  |
|  | 99 | NA |  |

# **Table S1. Characterization of voices by discipline**

| **Voice type** | **Account type** | **n** | **%** | **Natural sciences** | **Social Sciences** | **Human-ities** | **Not identify-able^a^** |
| --- | --- | --- | --- | --- | --- | --- | --- |
| *CENTRAL VOICES* | |  |  |  |  |  |  |
| *Institutional* | Administrative body | 4 | 0.6 | 0 | 0 | 0 | 4 |
| *Individual* | Representative | 11 | 1.8 | 0 | 0 | 0 | 11 |
|  | N_central_ | 15 | 2.4 | 0 | 0 | 0 | 15 |
| *DECENTRAL VOICES* | |  |  |  |  |  |  |
| *Institutional* | Department/institute  Division/team/lab  Administrative body  Student body | 31  18  4  6 | 5.0  2.9  0.6  1.0 | 13  12  0  0 | 12  3  3  1 | 4  2  0  1 | 2  1  1  4 |
|  | N_institutional_ | 59 | 9.5 | 25 | 19 | 7 | 8 |
| *Individual* | Professor  Researcher  Postdoc  PhD student  Student | 68  100  79  139  54 | 11.0  16.2  12.8  22.5  8.7 | 33  42  41  70  9 | 19  33  30  41  38 | 14  20  8  25  7 | 2  5  0  3  0 |
|  | N_individual_ | 440 | 71.1 | 195 | 161 | 74 | 10 |
|  | N_decentral_ | 499 | 80.6 | 220 | 180 | 81 | 18 |
| *FORMER VOICES* | |  |  |  |  |  |  |
| *Institutional* | Unit | - | - | - | - | - | - |
| *Individual* | Employee | 40 | 6.5 | 23 | 14 | 0 | 3 |
|  | Student | 39 | 6.2 | 4 | 20 | 6 | 9 |
|  | N_former_ | 79 | 12.8 | 27 | 34 | 6 | 12 |
| *NON IDENTIFIABLE* | | 26 | 4.2 | 5 | 11 | 3 | 7 |
|  | **N** | **619** | **100** | **252** | **225** | **90** | **52** |

*Note*. ^a^In some cases the discipline was not identifiable.

# **Table S2. Communities of voices based on mentions**

| **Community** | **Color** | **n** | **% of all ac-counts** | **Avg. in-degree** | **Median indegree** | **Avg. eigen-vector centrality** | **Median eigen-vector centrality** | **Share of connections (edges) to other commun-ities in %** |
| --- | --- | --- | --- | --- | --- | --- | --- | --- |
| Medicine | violet | 106 | 28.6 | 3.74 | 5 | 0.045 | 0.009 | 22.2 |
| Central voices | green | 103 | 27.8 | 3.73 | 1 | 0.042 | 0.003 | 35.0 |
| Social Sciences | blue | 56 | 15.1 | 7.4 | 6 | 0.104 | 0.063 | 15.6 |
| Humanities | yellow | 28 | 7.5 | 2.9 | 2 | 0.038 | 0.013 | 40.5 |
| Linguistics | orange | 28 | 7.5 | 2.4 | 1 | 0.031 | 0.004 | 30.4 |
| Economy | red | 23 | 6.2 | 3.0 | 2 | 0.025 | 0.005 | 31.4 |

*Note*. Indegree measures the amount of edges directed towards a node. In our case, accounts with a high indegree are accounts which are mentioned by many other accounts. Eigenvector centrality is a measure of influence of nodes (accounts) in connected networks. It is a relative score and determines to what extent a node is connected to other influential nodes. In our case, accounts with a high eigenvector are mentioned by many accounts who themselves are mentioned often by other accounts.

# **Table S3. Comparison of topics between official voices and all the multiple voices**

| **Topical  areas** | **Topics** | **Tweets of**  **official voices^a^** | | | **Mentions of official voices**  **by all the multiple voices combined^b^** | | |
| --- | --- | --- | --- | --- | --- | --- | --- |
|  |  | **Absolute amount** | **Relative amount** | **Engagement** | **Absolute amount** | **Relative amount** | **Engagement** |
|  |  | **n** | **%** | **M** | **n** | **%** | **M** |
| Academia- related | Research | 506 | 45.4 | 10.8 | 723 | 32.5 | 12.5 |
|  | Events | 152 | 13.6 | 10.0 | 463 | 20.8 | 8.2 |
|  | Teaching | 30 | 2.7 | 8.7 | 88 | 4.0 | 17.4 |
|  | **Total** | 688 | 61.7 | 10.5 | 1274 | 57.3 | 11.3 |
| Organization-related | Personnel | 24 | 2.2 | 12.0 | 166 | 7.5 | 38.7 |
|  | Performance | 53 | 4.8 | 12.1 | 96 | 4.3 | 15.8 |
|  | Health services | 57 | 5.1 | 30.3 | 56 | 2.5 | 6.4 |
|  | Alumni | 14 | 1.3 | 7.6 | 54 | 2.4 | 3.2 |
|  | Responsibility | 68 | 6.1 | 9.8 | 53 | 2.4 | 13.6 |
|  | Finances | 22 | 2.0 | 11.8 | 45 | 2.0 | 5.5 |
|  | Politics &  economy | 29 | 1.4 | 7.3 | 30 | 1.4 | 10.0 |
|  | Culture & sports | 27 | 2.4 | 13.2 | 21 | 0.9 | 8.7 |
|  | HEI politics & governance | 12 | 1.1 | 11.2 | 15 | 0.7 | 7.8 |
|  | **Total** | 306 | 27.4 | 14.4 | 537 | 24.2 | 18.7 |
| Other | Other (e.g., trivia, personal communication) | 121 | 10.9 | 13.6 | 392 | 17.6 | 4.7 |
| **Total** |  | **1’115** | **100** | **11.9** | **2’222** | **100** | **11.8** |

*Note.* ^a^ We present the findings aggregated for both accounts, as there are only few differences. In a few tweets, the topic was not identifiable or not applicable. ^b^ The right column is based on the findings shown in table 4 in the manuscript. Engagement metrics aggregate retweets and likes.

# **References**

Davies, S. R., & Horst, M. (2016). *Science communication: Culture, identity and citizenship.* Springer.

Fürst, S., Vogler, D., Schäfer, M. S., & Sörensen, I. (2021). Media Representations of Academia: Mapping and Typologizing News Coverage of All Swiss Higher Education Institutions. *International Journal of Communication, 15*, 3600–3620. <https://doi.org/10.5167/uzh-207345>

Fürst, S., Volk, S. C., Schäfer, M. S., Vogler, D., & Sörensen, I. (2022). Assessing changes in the public communication of higher education institutions: A survey of leaders of Swiss universities and colleges. *Studies in Communication Sciences, 22*(3), 515–534. <https://doi.org/10.24434/j.scoms.2022.03.3489>

Sörensen, I., Fürst, S., Vogler, D., & Schäfer, M. S. (2023). Higher education institutions on Facebook, Instagram, and Twitter. *Media and Communication, 11*(1), 264–277. <https://doi.org/10.17645/mac.v11i1.6069>

Vogler, D. (2020). Analyzing reputation of Swiss universities on Twitter–The role of stakeholders, content and sources. *Corporate Communications: An International Journal, 25*(3), 429–445. <https://doi.org/10.1108/CCIJ-04-2019-0043>
